# Reconstructing rearrangement phylogenies of natural genomes

**DOI:** 10.1186/s13015-025-00279-5

**Published:** 2025-06-07

**Authors:** Leonard Bohnenkämper, Jens Stoye, Daniel Doerr

**Affiliations:** 1 Faculty of Technology, Bielefeld University, Universitätsstraße 25, 33615 Bielefeld, NRW Germany; 2 Center for Biotechnology (CeBiTec), Bielefeld University, Universitätsstraße 25, 33615 Bielefeld, NRW Germany; 3 Department for Endocrinology and Diabetology, Medical Faculty, Heinrich Heine University Düsseldorf, University Hospital Düsseldorf, Moorenstr. 5, 40225 Düsseldorf, NRW Germany; 4 German Diabetes Center (DDZ), Leibniz Institute for Diabetes Research Germany, Auf’m Hennekamp 65, 40225 Düsseldorf, NRW Germany; 5 Center for Digital Medicine, Heinrich Heine University Düsseldorf, Moorenstr. 5, 40225 Düsseldorf, NRW Germany

**Keywords:** Genome rearrangement, Ancestral reconstruction, Small parsimony, Integer linear programming, Double-cut-and-join

## Abstract

**Background:**

We study the classical problem of inferring ancestral genomes from a set of extant genomes under a given phylogeny, known as the *Small Parsimony Problem* (SPP). Genomes are represented as sequences of oriented markers, organized in one or more linear or circular chromosomes. Any marker may appear in several copies, without restriction on orientation or genomic location, known as the *natural genomes* model. Evolutionary events along the branches of the phylogeny encompass large scale rearrangements, including segmental inversions, translocations, gain and loss (DCJ-indel model). Even under simpler rearrangement models, such as the classical breakpoint model without duplicates, the SPP is computationally intractable. Nevertheless, the SPP for natural genomes under the DCJ-indel model has been studied recently, with limited success.

**Methods:**

Building on prior work, we present a highly optimized ILP that is able to solve the SPP for sufficiently small phylogenies and gene families. A notable improvement w.r.t. the previous result is an optimized way of handling both circular and linear chromosomes. This is especially relevant to the SPP, since the chromosomal structure of ancestral genomes is unknown and the solution space for this chromosomal structure is typically large.

**Results:**

We benchmark our method on simulated and real data. On simulated phylogenies we observe a considerable performance improvement on problems that include linear chromosomes. And even when the ground truth contains only one circular chromosome per genome, our method outperforms its predecessor due to its optimized handling of the solution space. The practical advantage becomes also visible in an analysis of seven *Anopheles* taxa.

## Introduction

The *Small Parsimony Problem* (SPP) is a general optimization problem in phylogenetics that aims at annotating the internal vertices of a given phylogenetic tree $T = (V,E)$ whose leaves are already annotated, such that the total *tree distance* $d_T = \sum _{(A,B) \in E} d(A,B)$ is minimized. Here, *d*(*A*, *B*) is a function returning the distance between the annotations of any two vertices *A* and *B* of the phylogenetic tree. Traditional tree annotations may be DNA or protein sequences, while more recently, with the emergence of phylogenomic studies, also complete genomes, often in form of so-called *marker sequences* may be used.

Distance functions for marker sequences usually consider rearrangements and content-modifying operations on the elements of the sequences. A useful general-purpose distance in genome rearrangement is based on the *DCJ-indel* model. Conceived by Braga et al. [1] as an extension of the Double-Cut-and-Join model by Yancopoulos et al. [2], operations in the DCJ-indel model are either genomic rearrangements, modeled by a double cut and subsequent joining of the so created ends (*DCJ*), or segmental gains and losses of arbitrary length (*indels*).

When each marker occurs not more than once per genome, calculating the DCJ-indel distance between two genomes is polynomial [1]. However, on genomes with unrestricted distributions of markers, also called *natural genomes*, calculating the DCJ-indel distance is NP-hard. Nonetheless, efficient ILP solutions exist, such as *ding* [3].

The first attempt to generalize this method from the pairwise genomic distance to the phylogenomic SPP under the DCJ-indel model was an ILP by Doerr and Chauve [4], called *SPP-DCJ*. They did so by solving a generalized problem, in which – as a result of some pre-processing – adjacencies in ancestral genomes could be absent or present, and in the latter case they may be assigned a weight that would be taken into consideration during optimization. One major issue in this generalization was ding’s use of *caps*, telomeric markers that need to be matched during optimization and for which the solution space is superexponential [5]. Doerr and Chauve went to great lengths to limit the effect of this additional solution space, but were ultimately not able to completely remove it from their solution.

The ILP solution presented in this manuscript combines a recent reformulation of the DCJ-indel model that allows one to forego the matching of caps [6] with the basic modeling of SPP pioneered by SPP-DCJ. We additionally resolve another issue described in [4], which is the dependence of SPP-DCJ on previously known candidates for circular singletons, for each of which SPP-DCJ creates a number of constraints and variables. Since the number of circular singleton candidates in the worst case is exponential in the number of non-telomeric extremities, the worst case size of SPP-DCJ is exponential as well. While this problem may be less relevant when given few, refined candidate adjacencies for ancestors, our ILP is the first to solve the SPP for natural genomes under the DCJ-indel model while remaining of polynomial size w.r.t. any input data.

In practice, SPP, also known as small *phylogeny* problem, is central to many methods for ancestral genome reconstruction [7]. For instance, SPP-DCJ [4] is part of the AGO framework [8]. Other methods, such as GASTS [9] and MGRA [10] approach SPP by iteratively constructing median genomes. The genome median problem asks to construct one ancestral genome to $n \ge 3$ given genomes, a nevertheless NP-hard problem for which these and most other methods resort to heuristic or approximate solutions [9–11]. Algorithmic innovations based on ILPs [3, 6, 12] made it possible to compute exact solutions in practical applications. For instance, Frolova *et al.* [13] employ DING [3] in the calculation of pairwise DCJ indel distances to study phylogenetic relationships of pathogenic plasmids.

The remainder of the manuscript is organized as follows. In Section "Preliminaries", we give basic definitions and previous results needed to derive our algorithm. In Section "A new method", we explain the fundamental features of our method (Subsections "Capping-free model" and "On linearizability") before presenting the ILP in Subsection "A new ILP formulation" and detailing further methods of pre-processing to tighten the solution space in Subsection "Pre-processing". We evaluate the performance of our method in Section "Evaluation" and discuss our overall findings in Section "Discussion".

## Preliminaries

For the purposes of this work, we use the abstraction to describe genomes as sequences of oriented markers. A *(genomic) marker* $g = (g^\text {t}, g^\text {h})$ is a universally unique entity consisting of *marker extremities* tail of *g*, denoted by $g^\text {t}$, and head of *g*, denoted by $g^\text {h}$.

The structure of a genome can be described via its adjacencies. An adjacency $\{f^x,g^y\}$ (with $x,y \in \{\text {t},\text {h}\}$) describes that markers *f* and *g* are neighbors on the same chromosome and oriented, such that extremities $f^x$ and $g^y$ are adjacent. For ease of notation we also write $f^xg^y$ for an adjacency. Note that adjacencies can be read in either direction, i.e. $g^yf^x$ is the same as $f^xg^y$.

For the sake of a simpler formulation of the theory, we aim for each extremity to be part of some adjacency. In order to accomplish this, we use additional extremities modeling the ends of linear chromosomes, called *telomeres*. A *telomere* $t^\circ$ is a universally unique entity encompassing a single telomeric extremity denoted by “$\circ$”. A genome can then be described as a graph as follows.

### Definition 1

A *genome* $\mathbb {A}$ is a graph with vertices $\mathcal {E}(\mathbb {A})\cup \mathcal {T}(\mathbb {A})$, namely its marker extremities $\mathcal {E}(\mathbb {A})$ and telomeric extremities $\mathcal {T}(\mathbb {A})$. The set of edges is $\mathcal {M}(\mathbb {A})\cup \mathcal {A}(\mathbb {A})$, namely its marker edges $\mathcal {M}(\mathbb {A})$ and adjacency edges $\mathcal {A}(\mathbb {A})$. This graph fulfills the following properties:

1. $\mathcal {M}(\mathbb {A})$ is a perfect matching on $\mathcal {E}(\mathbb {A})$ with $\mathcal {M}(\mathbb {A}) = \{\{m^t,m^h\} \mid \forall m^t,m^h\in \mathcal {E}(\mathbb {A})\}$, and
2. $\mathcal {A}(\mathbb {A})$ is a perfect matching on $\mathcal {E}(\mathbb {A})\cup \mathcal {T}(\mathbb {A})$.

**Fig. 1:**
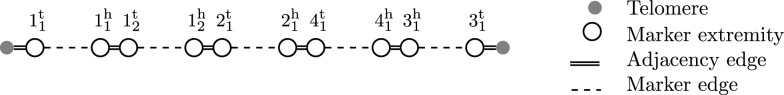
A genome of five markers $1_1$, $1_2$, $2_1$, $3_1$, $4_1$ on a single linear chromosome

An example of a genome is given in Fig. 1.

Because each marker is universally unique, in order to compare genomes we need to establish which markers are homologous. We model homology as an equivalence relation ($\equiv$), that is $m_a\equiv m_b$ for some markers $m_a\in \mathcal {M}(\mathbb {A})$, $m_b\in \mathcal {M}(\mathbb {B})$ and genomes $\mathbb {A},\mathbb {B}$. Note that this includes the case $\mathbb {A}=\mathbb {B}$, i.e. there can be homologous markers in the same genome (in-paralogs). The equivalence class of a marker *m*, denoted by [*m*], is called its *family*. If a marker *m* exists in $\mathbb {A}$, but has no equivalent in $\mathbb {B}$ or vice versa, we refer to *m* as *singular* w.r.t. $\mathbb {A},\mathbb {B}$.

Given the equivalence relation on markers, one can easily derive an equivalence relation on extremities, namely $m_a^\text {t}\equiv m_b^\text {t}$ and $m_a^\text {h}\equiv m_b^\text {h}$ if and only if $m_a\equiv m_b$. For this derived equivalence we have $m_a^\text {h}\not \equiv m_b^\text {t}$ for all $m_a,m_b$. We call extremities *singular* if and only if their corresponding marker is singular. One can visualize such an equivalence relation for two genomes $\mathbb {A},\mathbb {B}$ using the Capping-Free Multi-Relational Diagram as defined in Definition 2.

### Definition 2

Given two genomes $\mathbb {A},\mathbb {B}$ and a homology ($\equiv$), the *Capping-Free Multi-Relational Diagram (CFMRD)* is a graph $\mathcal {CFMRD}(\mathbb {A},\mathbb {B},\equiv )=(\mathcal {E}\cup \mathcal {T},{E}_\text {adj}\cup {E}_\text {self}\cup {E}_{\text {ext}})$ with $\mathcal {E}=\mathcal {E}(\mathbb {A})\cup \mathcal {E}(\mathbb {B})$, $\mathcal {T}=\mathcal {T}(\mathbb {A})\cup \mathcal {T}(\mathbb {B})$, adjacency edges ${E}_\text {adj}=\mathcal {A}(\mathbb {A})\cup \mathcal {A}(\mathbb {B})$, self edges ${E}_\text {self}=\{m \in \mathcal {M}(\mathbb {A}) \cup \mathcal {M}(\mathbb {B}) \mid m \text { singular w.r.t.\ }\mathbb {A},\mathbb {B}\}$ and extremity edges ${E}_{\text {ext}}= \{ \{u,v\} \mid u \in \mathcal {E}(\mathbb {A}), v \in \mathcal {E}(\mathbb {B}), u\equiv v\}$.

**Fig. 2:**
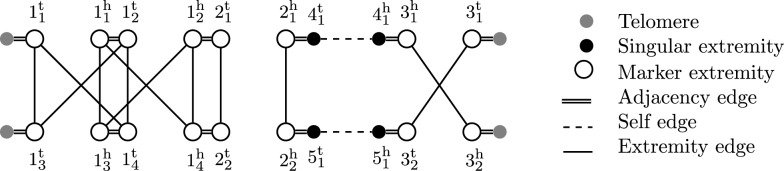
Capping-Free Multi-Relational Diagram for two genomes on an unresolved homology ($\equiv _1$) with families $\{1_1,1_2,1_3,1_4\},$ $\{2_1,2_2\},\{3_1,3_2\},\{4_1\},\{5_1\}.$

An example of a genome is given in Fig. 2.

**Fig. 3:**
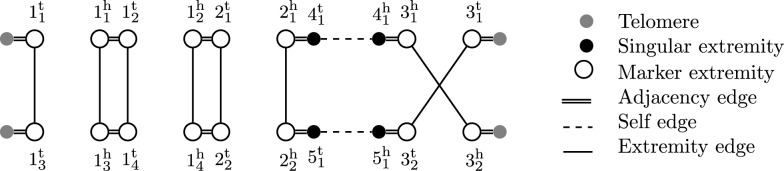
Acrshort*cfmrd for the two genomes of Fig. 2 on a resolved homology ($\equiv _2$) with families $\{1_1,1_3\}$, $\{1_2,1_4\}$, $\{2_1,2_2\}$, $\{3_1,3_2\}$, $\{4_1\}$, $\{5_1\}$. Note that ($\equiv _2$) is a matching on ($\equiv _1$)

An established way to compare two genomes on a structural level is the *rearrangement distance*. The rearrangement distance of two genomes $\mathbb {A},\mathbb {B}$ is defined as the minimum number of operations needed to transform $\mathbb {A}$ into $\mathbb {B}$ with operations restricted to a certain model (such as DCJ-indel). When ($\equiv$) maps each marker of genome $\mathbb {A}$ to at most one marker of genome $\mathbb {B}$, calculating the rearrangement distance between $\mathbb {A}$ and $\mathbb {B}$ is typically easy. We refer to such a homology as *resolved*. More formally, a homology is resolved if for each genome $\mathbb {A}$ and marker $m\in \mathcal {M}(\mathbb {A})$ the family of *m* contains only itself, i.e. $[m]\cap \mathcal {M}(\mathbb {A})=\{m\}$. On these homologies, $\mathcal {CFMRD}(\mathbb {A},\mathbb {B},\equiv )$ consists only of simple cycles and simple paths. An example of a CFMRD on a resolved homology is shown in Fig. 3.

With a resolved homology, the DCJ-indel distance can be calculated easily by just counting different types of components in the CFMRD. For the purpose of this counting, we ignore self edges. We write *c* for the number of cycles and $p_{ab}$ (resp. $p_{aa}$, resp. $p_{bb}$) for the number of paths that start in $\mathbb {A}$ and end in $\mathbb {B}$ (resp. start in $\mathbb {A}$ and end in $\mathbb {A}$, resp. start in $\mathbb {B}$ and end in $\mathbb {B}$). Since the graph is undirected, we canonize their labels by reading paths from $\mathbb {A}$ to $\mathbb {B}$. When the vertex the path starts or ends in is a telomere of $\mathbb {A}$ (resp. $\mathbb {B}$), we write *A* (resp. *B*) in uppercase. When the path ends because the only way to continue it would be a self edge (note that we ignore self edges here), we write *a* (resp. *b*) in lowercase. When a path starts and ends in the same genome, we read it from telomere to singular extremity (note that in all other cases, the label is symmetric).

For example, the CFMRD of Fig. 3 has $c=2$, $p_{AB}=1$ (path $t^\circ ,1_1^\text {t}, 1_3^\text {t},t^\circ$), $p_{ab}=1$ (path $4_1^\text {t},2_1^\text {h},2_2^\text {h},5_1^\text {t}$), $p_{aB}=1$ (path $4_1^\text {h},3_1^\text {h},3_2^\text {h},t^\circ$) and $p_{Ab}=1$ (path $t^\circ ,3_1^\text {t},3_2^\text {t},5_1^\text {h}$).

There is one case, in which we need to consider self edges, namely *circular singletons*. Circular singletons are cycles that consist only of adjacency and self edges. We denote their number by *s*. For a more in-depth explanation of these terms, the interested reader is referred to [6]. Using these terms, the following formula can be used.

### Theorem 1

(adapted from [6]) For two genomes $\mathbb {A},\mathbb {B}$ and a resolved homology (${{\mathop {\equiv }\limits ^{\star }}}$), the DCJ-indel distance is

$$\begin{aligned} \bar{d}_\mathrm {DCJ-ID}(\mathbb {A}, \mathbb {B}, {{\mathop {\equiv }\limits ^{\star }}}) \, = \, n -c + \left\lceil \frac{p_{a b} + \max (p_{A a},p_{a B}) + \max (p_{A b},p_{B b}) - p_{A B}}{2}\right\rceil + s \end{aligned}$$

with *n* the number of *matched markers*, $n = |\{(m_a,m_b)\in \mathcal {M}(\mathbb {A})\times \mathcal {M}(\mathbb {B}) \mid m_a{{\mathop {\equiv }\limits ^{\star }}}m_b\}|$.

This formula holds because it is equivalent to previously proven distance formulas under the DCJ-indel model, however it can also be derived independently. Details are explained in [6]. To paraphrase the results there, it is shown that two genomes are equal if and only if their CFMRD consists of only *c* cycles and $p_{AB}$ paths between telomeres of both genomes with $n=c+\frac{p_{AB}}{2}$. Additionally, for each DCJ or indel operation the formula of Theorem 1 changes by at most 1. These two facts combined yield the formula as a lower bound. Additionally [6] contains an algorithm transforming $\mathbb {A}$ into $\mathbb {B}$ using DCJ and indel operations that is able to reach this lower bound, proving it is a formula for the rearrangement distance under the DCJ-indel model.

When the homology is not resolved, we need to refine the homology to be resolved. We call such a refinement a *matching*. More formally, a matching (${{\mathop {\equiv }\limits ^{\star }}}$) on ($\equiv$) is a resolved homology, such that $m_a{{\mathop {\equiv }\limits ^{\star }}}m_b\implies m_a\equiv m_b$.

Since allowing for arbitrary matchings can lead to an excess of indels in the sorting scenario, we restrict ourselves to the maximum matching model. A matching $({{\mathop {\equiv }\limits ^{+}}})$ is *maximum* w.r.t. $\mathbb {A},\mathbb {B}$ if a maximum amount of markers in $\mathbb {A}$ has a homolog in $\mathbb {B}$ and vice versa.

### Definition 3

Given homology ($\equiv$), the DCJ-indel distance between $\mathbb {A}$ and $\mathbb {B}$ under the maximum matching model is

$$\begin{aligned} d_{\mathrm {DCJ-ID}}(\mathbb {A},\mathbb {B},\equiv ) \, = \, \min _{({{\mathop {\equiv }\limits ^{+}}}) \mathrm {\ maximum\ matching\ on\ } (\equiv )} \bar{d}_\mathrm {DCJ-ID}(\mathbb {A},\mathbb {B},{{\mathop {\equiv }\limits ^{+}}}). \end{aligned}$$

When reconstructing a phylogeny, only extant genomes are known, that is, there is no definitive information about the adjacencies at the inner nodes. In order to capture this uncertainty, a typical approach is to generate a large set of candidate adjacencies at each inner node that very likely will include the correct ones. Such a set can be viewed as a *degenerate genome*, which however may contain multiple conflicting adjacencies, such as *ab* and *ac* with $b\ne c$. (In a normal genome this cannot occur, as the matching requirement ensures that there is only one adjacency that involves *a*.) More formally, a degenerate genome $\mathbb {D}$ is a graph $(\mathcal {E}(\mathbb {D})\cup \mathcal {T}(\mathbb {D}), \mathcal {M}(\mathbb {D})\cup \mathcal {A}(\mathbb {D}))$ that fulfills only Property 1 of Definition 1.

**Fig. 4:**
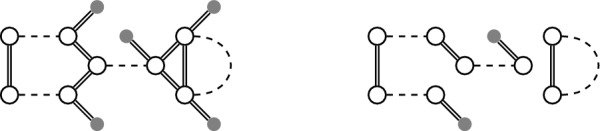
Left: A degenerate genome. Right: A linearization of it

All possible ancestors at a certain node in the phylogeny are then built from disambiguations of these conflicting adjacencies. We call these possible ancestors *linearizations*. A linearization of a degenerate genome $\mathbb {D}$ is a genome $\mathbb {A}$, such that $\mathcal {E}(\mathbb {A})=\mathcal {E}(\mathbb {D})$, $\mathcal {T}(\mathbb {A}) \subseteq \mathcal {T}(\mathbb {D})$, $\mathcal {M}(\mathbb {A})=\mathcal {M}(\mathbb {D})$ and $\mathcal {A}(\mathbb {A})\subseteq \mathcal {A}(\mathbb {D})$. If such a linearization exists, we call $\mathbb {D}$ *linearizable*. We give an example of a linearizable degenerate genome and one of its linearizations in Fig. 4. Note that each genome is also a degenerate genome with precisely one linearization, namely itself.

We can then formulate the problem we are considering in this paper as finding linearizations of all (degenerate) genomes in the phylogeny, such that the sum of all DCJ-indel distances in the tree is minimized. Optionally, we also allow to put weights on the adjacencies and take these into account during the minimization.

### Problem 1

(Weighted Small Parsimony Linearization Problem) Given a phylogeny $T=(V,E)$, a homology ($\equiv$), a weighting function *w* for adjacencies, and a parameter $\alpha \in [0,1]$, find a linearization $\mathbb {L}_i$ for each (degenerate) genome $\mathbb {D}_i$ in *T*, such that

1 $$\begin{aligned} \sum _{(\mathbb {D}_i,\mathbb {D}_k)\in E} \left( \alpha \, d_{\mathrm {DCJ-ID}}(\mathbb {L}_i,\mathbb {L}_k,\equiv ) \, + \, (\alpha -1) \sum _{ab\in \mathcal {A}(\mathbb {L}_i)\cup \mathcal {A}(\mathbb {L}_k)} w(ab)\right) \end{aligned}$$

is minimized.

Because the pairwise comparison of (non-degenerate) natural genomes is already NP-hard, the Weighted Small Parsimony Linearization Problem is NP-hard as well. Doerr and Chauve’s algorithm SPP-DCJ, which solves Problem 1, is therefore formulated as an ILP. Thus, we formulate our improved algorithm in Section 3.3 as an ILP as well.

## A new method

### Capping-free model

The previous solution by Doerr and Chauve [4] was based on a different graph structure, namely the Capped Multi-Relational Diagram (CMRD).. The CMRD differs from the CFMRD in the way it treats telomeres. In the CMRD of two genomes $\mathbb {A}$ and $\mathbb {B}$ there exist additional extremity edges between each telomere of $\mathbb {A}$ and each telomere of $\mathbb {B}$, leading to additional $|\mathcal {T}(\mathbb {A})| \cdot |\mathcal {T}(\mathbb {B})|$ extremity edges.

When calculating the DCJ-indel distance using the CMRD, one must not only determine the resolved homology, but also a matching between telomeres, that is, on $\mathcal {T}(\mathbb {A}) \times \mathcal {T}(\mathbb {B})$. As identified in [5], this leads to a superexponential increase of the solution space. As our new method is based on the CFMRD, we can use the formula of Theorem 1 and thus avoid such an increase in the solution space.

### On linearizability

**Fig. 5:**
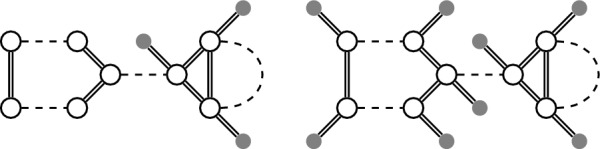
Left: This degenerate genome is not linearizable because of missing telomeres. Right: The genome becomes linearizable when adding telomeres. One linearization is that of Fig. 4

It is vital for our method that the degenerate genomes in the phylogeny are linearizable (see Problem 1). However, not all degenerate genomes are linearizable (see Fig. 5). Moreover, not all methods used to infer candidate adjacencies for ancestors guarantee this requirement. In particular DeCoSTAR [14], a method for inferring ancestral genomes that is integrated together with SPP-DCJ into a larger reconstruction workflow detailed in [8], generates conflicting ancestral adjacencies.

As far as we are aware, no algorithms testing for linearizability in polynomial time exist as of yet. However, we give an algorithm here that is able to generate a linearization if one exists, by proxy solving the testing problem.

Recall that $\mathcal {T}(\mathbb {D})$ are the telomeres and $\mathcal {E}(\mathbb {D})$ are the extremities of a degenerate genome $\mathbb {D}$. We are interested in finding a matching *M* on the adjacencies $\mathcal {A}(\mathbb {D})$ of $\mathbb {D}$, such that each extremity is part of exactly one edge in *M*. This is equivalent to the linearization problem as any telomeres not part of the matching can then be removed and one obtains a genome.

To see how we are able to determine such a matching, consider the weight function *w* that assigns to each adjacency edge $\{u,v\}\in \mathcal {A}(\mathbb {D})$ the number of extremities incident to it: $w(\{u,v\}) = |\{u,v\} \cap \mathcal {E}(\mathbb {D})|$.

#### Lemma 1

$\mathbb {D}$ is linearizable if and only if a maximum weight matching *M* on the weighted graph $\big (\mathcal {T}(\mathbb {D})\cup \mathcal {E}(\mathbb {D}),\mathcal {A}(\mathbb {D}),w\big )$ has total weight $|\mathcal {E}(\mathbb {D})|$.

#### Proof

Note that there are no edges $\{u,v\}$ with both $u,v\in \mathcal {T}(\mathbb {D})$.

Assume a matching $M_S$ that covers the subset $S\subseteq \mathcal {E}(\mathbb {D})$. We further subdivide *S* into the disjoint sets $S_1$ and $S_2$. $S_1$ contains all vertices $v\in S$ that are matched with a telomere, that is $(v,u)\in M_S$ with $u\in \mathcal {T}(\mathbb {D})$. $S_2$ contains the vertices that are matched with another extremity (note that for $v\in S_2$ and $(v,u)\in M_S$ follows $u\in S_2$). Since there are no edges between telomeres directly, the total weight of $M_S$ is

$$\begin{aligned} \sum _{\{u,v\}\in M_S} w(\{u,v\})&= \sum _{\{u,v\}\in M_S, u \text { or }v \in S_1} w(\{u,v\}) + \sum _{\{u,v\}\in M_S,u,v\in S_2} w(\{u,v\})\\ &= |S_1| + 2 \frac{|S_2|}{2} = |S| \end{aligned}$$

We thus see that a matching has weight *k* if and only if it covers a subset of $\mathcal {E}(\mathbb {D})$ of size *k*. The claim of the lemma follows by noting that a matching can have at most weight $|\mathcal {E}(\mathbb {D})|$ and that if such a matching $M_\mathcal {E}$ exists, we can use $M_\mathcal {E}$ as the adjacencies of the linearization of $\mathbb {D}$. $\square$

Using Lemma 1, we can either find that there is no linearization or determine one using a standard maximum weight matching algorithm for any degenerate genome $\mathbb {D}$.

While we can test whether genomes are linearizable using this maximum weight matching algorithm, previous versions of SPP-DCJ modified the given degenerate genomes by adding telomeres, such that they are guaranteed to be linearizable, which may still be desirable on noisy data (see Subsection 3.2.2). We detail these methods briefly in the following subsections.

#### Local guarantees

The first method of guaranteeing linearizability relies on the following lemma.

##### Lemma 2

A perfect matching $M \subseteq \mathcal {A}(\mathbb {D})$ in a degenerate genome $\mathbb {D}= (\mathcal {E}(\mathbb {D})\cup \mathcal {T}(\mathbb {D}), \mathcal {M}(\mathbb {D})\cup \mathcal {A}(\mathbb {D}))$ corresponds to a linearization of $\mathbb {D}$.

##### Proof

Observe that in the *M*-induced degenerate genome $\mathbb {D}' = (\mathcal {E}(\mathbb {D}) \cup \mathcal {T}(\mathbb {D}), \mathcal {M}(\mathbb {D}) \cup M)$ each node is incident to exactly one adjacency edge. Further each connected component corresponds to a linear component where both degree-one nodes correspond to telomeres, or a circular component where each node corresponds to a marker extremity. $\square$

However, the converse is not true: Since not all telomeric extremities must be covered, $\mathbb {D}$ may still be linearizable even if no perfect matching may be derived from $\mathbb {D}$.

In an earlier version of SPP-DCJ [4], a simple approach was introduced that complements each degenerate genome $\mathbb {D}$ with additional telomeres and telomeric adjacencies to ensure linearizability. To this end, $\mathbb {D}$ is decomposed into connected components that are independently tested. If the size of a component, i.e., the number of its vertices, is even, and it is either linear, circular, or fully connected, then it is considered as *locally linearizable*. Otherwise, each extremity *v* of the component is complemented with a telomere $t_v$, and a telomeric adjacency $\{v,t_v\}$ is added to the degenerate genome, ensuring that it is linearizable as a whole.

#### Allowing each extremity to be connected to a telomere

Given the uncertainty about inferred ancestral adjacencies, even when a component is locally linearizable, individual adjacencies of that component might still be wrongly inferred by the pre-processing and thus might be erroneously included in the linearization, simply because otherwise a linearization might not be possible.

In order to prevent this behavior, we offer a mode in which *each* extremity is connected to an (artificially introduced) telomere to reflect this uncertainty. In contrast to the method described above, we do this even in components with local guarantees.

This approach was previously practically unsound because of inefficient handling of telomeres. Now it may become the standard mode of operation, as it allows to find reasonable solutions in case of noisy input data, while the computational overhead introduced by the addition of the artificial telomeres remains moderate. We refer to this mode as the *safer linearization mode* in subsequent sections.

### A new ILP formulation

Algorithm 1 gives an overview of our method with additional tables detailing parts of the ILP.

In principle, our algorithm solves Problem 1 in the same way as SPP-DCJ [4], namely it determines linearizations while simultaneously computing the distances between nodes in the phylogeny with the objective of minimizing the total distance. However, for ease of readability, we separate the linearization and distance computation into two different subsections.

On the *global level*, the linearizations $\mathbb {L}_i$ are derived for each (degenerate) genome $\mathbb {D}_i$. On the *local level*, the resulting linearizations are compared to each other along the branches of the phylogeny. Each branch gives rise to a pairwise comparison by means of the CFMRD. In doing so, the selection of adjacencies of a derived genome is propagated from across CFMRDs, thus ensuring global consistency.

The main differences between our algorithm and that in [4] are found in the local level, as this is where the CFMRD plays a role.

#### Global level

The global level deals with the setting of adjacencies or telomeres of (ancestral) genomes. For each (marker or telomeric) extremity *v*, we determine its presence or absence with a binary variable ${\texttt {g}}_v$. For markers, the head extremity is present if and only if the tail extremity is (see Constraint C.01). Since there is often uncertainty about the precise copy number of markers in ancestral genomes, we allow user-determined bounds $(L_F^\mathbb {A},H_F^\mathbb {A})$ for the number of markers in each family *F* in ancestral genome $\mathbb {A}$ (C.02). If not specified, these bounds default to the maximum, requiring each marker to occur, that is they collapse to

$$\begin{aligned} (\texttt {C.01A}) \quad {\texttt {g}}_v = 1 \quad v\in \{m^t,m^h\} \text { for } m \in \mathcal {M}\text { with } L_{[m]}^\mathbb {A}= H_{[m]}^\mathbb {A}=|[m]\cap \mathcal {M}|. \end{aligned}$$

Each extremity present is then required to be part of exactly one (possibly telomeric) adjacency (C.03), which ensures a properly linearized genome.

#### Local level

The local level deals with each edge of the tree separately, making use of the CFMRD of the corresponding genome pair. Since this part is entirely local to the edge in question, we presume that each vertex $v_i$ of the CFMRD has a unique identifier among all other CFMRDs , making all its variables globally unique. In order to limit the range of the general variable $y_{v_i}$, we also assign each vertex a rank *i* that is local and unique only within the specific CFMRD . We map each extremity to its identifier for the global level by the function $\gamma$.

In order to compute decompositions of CFMRDs, we make use of a capping-free formulation for the computation of the pairwise DCJ indel distance derived in [6]. This formulation is based on the distance formula found in Theorem 1.

The formulation counts cycles ${\texttt {c}}_E$ as well as the six different types of paths relevant to Theorem 1, namely ${\texttt {p}}^{ab},{\texttt {p}}^{Aa},{\texttt {p}}^{aB},{\texttt {p}}^{Ab},{\texttt {p}}^{Bb},{\texttt {p}}^{AB}$. Each counting variable ${\texttt {p}}^X$ is set by summing up binary report variables ${\texttt {r}}^X_v$ that are set to 1 once per component on a specific vertex *v* (see Constraints C.09 to C.13 and C.18). These counters are then combined to the terms of the formula in Constraints C.14 to C.17 and C.04 to C.08. The constraints for ensuring the reporting variables being set correctly can be found in Tables 1, 2 and 3. For a complete description of this part of the ILP the interested reader is referred to [6].

**Table 1:** Shao-Lin-Moret constraints [12]

(C.27)	${\texttt {x}}_e={\texttt {x}}_d$	for all sibling edges *e*, *d*
(C.28)	${\texttt {y}}_{v_i} + j(1-{\texttt {x}}_{u_jv_i}) \ge {\texttt {y}}_{u_j}$	$\forall u_jv_i \in {E}_\text {adj}\cup {E}_{\text {ext}}$
	$j(1-{\texttt {x}}_{u_jv_i})\ge {\texttt {y}}_{u_j}$	$\forall u_jv_i \in {E}_\text {self}$
(C.29)	$i{\texttt {z}}_{v_i} \le {\texttt {y}}_{v_i}$	$\forall v\in \mathcal {E}\cup \mathcal {T}$

**Table 2:** Reporting for regular vertices

(C.30)	${\texttt {l}}_v \le 1 - {\texttt {x}}_{uv}$	$\forall uv \in {E}_\text {self},u\in \mathcal {E}(\mathbb {A})$
	${\texttt {l}}_v \ge {\texttt {x}}_{uv}$	$\forall uv \in {E}_\text {self},u\in \mathcal {E}(\mathbb {B})$
(C.31)	${\texttt {l}}_v \le {\texttt {l}}_u + (1-{\texttt {x}}_{uv})$	$\forall uv \in {E}_{\text {ext}}$
	${\texttt {l}}_u \le {\texttt {l}}_v + {\texttt {r}}_{uv}^{ab} + (1-{\texttt {x}}_{uv})$	$\forall uv\in E_{adj},u\in \mathcal {E}(\mathbb {A})$
	${\texttt {l}}_u \le {\texttt {l}}_v + (1-{\texttt {x}}_{uv})$	$\forall uv\in E_{adj},u\in \mathcal {E}(\mathbb {B})$
(C.32)	${\texttt {r}}_{v}^c \le {\texttt {z}}_v$	$\forall v \in \mathcal {E}(\mathbb {A})$
(C.33)	${\texttt {r}}_{u}^{ab} \le {\texttt {x}}_{uv}$	$\forall uv\in {E}_\text {self},u\in \mathcal {E}(\mathbb {A})$

**Table 3:** Reporting for telomeres

(C.34)	${\texttt {l}}_v = 0$	$\forall v \in \mathcal {T}(\mathbb {A})$
	${\texttt {l}}_v = 1$	$\forall v \in \mathcal {T}(\mathbb {B})$
(C.35)	${\texttt {l}}_u \le {\texttt {l}}_v + {\texttt {r}}_{v}^{AB} + {\texttt {r}}_{v}^{Ab} + (1-{\texttt {x}}_{uv})$	$\forall uv\in {E}_\text {adj}, v \in \mathcal {T}(\mathbb {A})$
	${\texttt {l}}_u \le {\texttt {l}}_v + {\texttt {r}}_{u}^{aB} + (1-{\texttt {x}}_{uv})$	$\forall uv\in {E}_\text {adj}, u\in \mathcal {T}(\mathbb {B})$
(C.36)	${\texttt {r}}_{v}^{AB} \le {\texttt {z}}_v$	$\forall v \in \mathcal {T}(\mathbb {A})$
(c.37)	$1 -{\texttt {y}}_v \le {\texttt {r}}^{Ab}_{v} + {\texttt {r}}^{Aa}_v$	$v \in \mathcal {T}(\mathbb {A})$
	$1 -{\texttt {y}}_v \le {\texttt {r}}^{aB}_{v} + {\texttt {r}}^{Bb}_v$	$v \in \mathcal {T}(\mathbb {B})$
(C.38)	${\texttt {y}}_{v_i} \le i(1-{\texttt {r}}_{v}^{R})$	$v\in \mathcal {T}(\mathbb {A}),R\in \{Ab, Aa\}$
	${\texttt {y}}_{v_i} \le i(1-{\texttt {r}}_{v}^{R})$	$v\in \mathcal {T}(\mathbb {B}),R\in \{aB, Bb\}$
(C.39)	${\texttt {r}}_{v}^{AB} \le {\texttt {l}}_u + (1-x_{uv})$	$\forall uv\in {E}_\text {adj}, v \in \mathcal {T}(\mathbb {A})$
	${\texttt {r}}_{v}^{Ab} \le {\texttt {l}}_u + (1-x_{uv})$	$\forall uv\in {E}_\text {adj}, v \in \mathcal {T}(\mathbb {A})$
	${\texttt {r}}_{v}^{aB} \le 1-{\texttt {l}}_u + (1-x_{uv})$	$\forall uv\in {E}_\text {adj}, v \in \mathcal {T}(\mathbb {B})$

We make only few major changes in our local section w.r.t. the ILP described in [6]. Firstly, we determine whether an adjacency edge *e* is set (${\texttt {x}}_e=1$) by “inheriting” this value from the linearization generated in the global section (see C.21) of the corresponding adjacency. Secondly, we allow only vertices that are part of the linearized genome (${\texttt {g}}_v=1$) to contribute to the count of components that decrease the formula (${\texttt {z}}_v=1$), see C.22. To enforce the Maximum matching model, for any family we allow self edges only in one of the two genomes (C.23). If it is clear from the bounds, in which genome the family will be overrepresented, the self edges in the underrepresented genome can be removed and the constraint can be dropped.

Due to the fact that ancestral genomes may be degenerate, the number of possible circular singletons can be as large as the number of possible circular chromosomes. Listing all candidates, such as is done in [6] and in SPP-DCJ [4], leads to a combinatorial explosion on certain input data. Particularly, when all possible adjacencies are present in the degenerate genome, any non-empty subset of singular markers can form a circular singleton. A lower bound on the number of candidates is therefore $$\sum _{i=1}^{|{E}_\text {self}|} \left( {\begin{array}{c}|{E}_\text {self}|\\ i\end{array}}\right) = 2^{|{E}_\text {self}|}-1$$. To avoid an exponential worst case size of our ILP, we use a new technique for counting circular singletons without listing all candidates when the number of candidates is larger than a given (polynomial) threshold, which we arbitrarily set at twice the number of self edges. The constraints for this technique are listed in Table 4 and described in the following.

**Algorithm 1:**
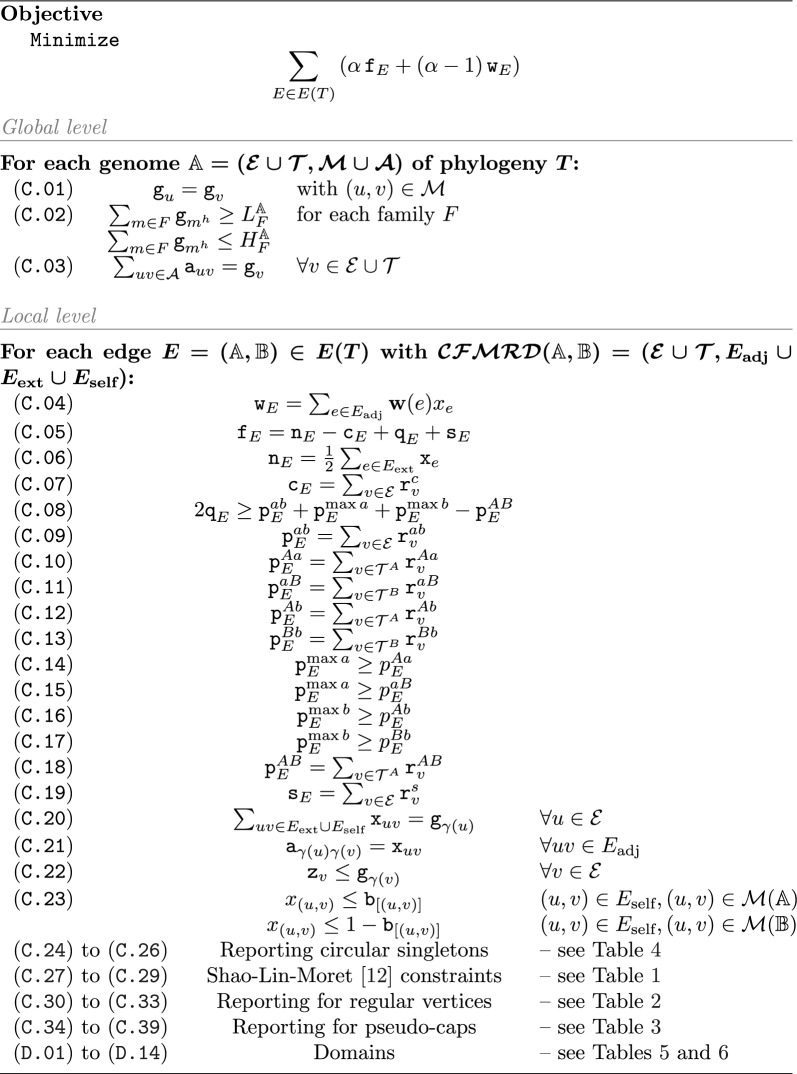
Capping-free Small Parsimony

**Table 4:** Reporting circular singletons

(C.24)	${\texttt {d}}_u+{\texttt {d}}_v + {\texttt {x}}_{uv}\le 2$	$\forall uv \in {E}_\text {adj}\cup {E}_\text {self}$
	${\texttt {d}}_u + {\texttt {d}}_v - {\texttt {x}}_{uv} \ge 0$	$\forall uv \in {E}_\text {adj}\cup {E}_\text {self}$
(C.25)	${\texttt {w}}_u = {\texttt {w}}_v$	$\forall uv \in {E}_\text {self}$
(C.26)	$K (1- {\texttt {x}}_{uv} + {\texttt {r}}^{s}_{u} + {\texttt {r}}^{s}_{v}) + {\texttt {w}}_v \ge {\texttt {w}}_u + {\texttt {d}}_v - {\texttt {d}}_u$	$\forall uv\in {E}_\text {adj}$

A circular singleton manifests in the graph as a cycle of alternating adjacency and indel edges. The idea of the technique is to have a general integer variable ${\texttt {w}}$ that is required to increase at each adjacency edge in a walk of the cycle. There must then be one point in the walk in which it decreases again. Detecting this, one can then report a circular singleton. For this to work, the walk needs a direction. This is accomplished by annotating the vertices with a binary variable ${\texttt {d}}_v$ that “flips” across each pair of connected vertices (see C.24). We then require ${\texttt {w}}$ to be the same for vertices connected by an indel edge (see C.25) and for it to increase by 1 in the direction of the vertex that has ${\texttt {d}}_v=1$ (see C.26). We require this except when vertices are not connected ($1-{\texttt {x}}_{uv}=0$) or when reporting a circular singleton (${\texttt {r}}^s_u=1$ or ${\texttt {r}}^s_v=1$). In this case, the constraint is automatically fulfilled by adding the maximum length of circular singletons *K* to the left hand side of the inequation.

**Table 5:** Domains - global level

(D.01)	${\texttt {g}}_v\in \{0,1\}$	for each genome $\mathbb {X}$, $\forall v \in \mathcal {E}(\mathbb {X})\cup \mathcal {T}(\mathbb {X})$
(D.02)	${\texttt {f}}_E,{\texttt {n}}_E,{\texttt {c}}_E,{\texttt {s}}_E\in \mathbb {N}_0$	$\forall E\in E(T)$
(D.03)	${\texttt {p}}^{xy}_E,p^{\max a}_E,p^{\max b}_E\in \mathbb {N}_0$	$\forall E\in E(T)\, \forall x,y\in \{A,B,a,b\},x\ne y$
(D.04)	$q_E\in \mathbb {Z}$	$\forall E\in E(T)$
(D.05)	${\texttt {w}}_E\in \mathbb {R}$	$\forall E\in E(T)$

**Table 6:** Domains - local level. For each edge $(\mathbb {A},\mathbb {B}) \in E(T)$ with $\mathcal {CFMRD}(\mathbb {A},\mathbb {B}) = (\mathcal {E}\cup \mathcal {T}, E_\text {all})$ with $E_\text {all} = {E}_\text {adj}\cup {E}_{\text {ext}}\cup {E}_\text {self}$:

(D.06)	${\texttt {x}}_{e}\in \{0,1\}$	$\forall e\in E_\text {all}$
(D.07)	${\texttt {y}}_{v_i}\in \{0,...,i\}$	$v_i\in \mathcal {E}\cup \mathcal {T}$
(D.08)	${\texttt {z}}_{v},l_v\in \{0,1\}$	$v\in \mathcal {E}\cup \mathcal {T}$
(D.09)	${\texttt {d}}_v\in \{0,1\}$	$v\in \mathcal {E}$
(D.10)	${\texttt {w}}_v\in \mathbb {N}_0$	$v\in \mathcal {E}$
(D.11)	$r^{ab}_v\in \{0,1\}$	$\forall v\in \mathcal {E}(\mathbb {A})$
(D.12)	$r^{Aa}_v,r^{Ab}_v,r^{AB}_v\in \{0,1\}$	$\forall v\in \mathcal {T}(\mathbb {A})$
(D.13)	$r^{aB}_v,r^{Bb}_v\in \{0,1\}$	$\forall v \in \mathcal {T}(\mathbb {B})$
(D.14)	$b_f\in \{0,1\}$	for each family *f*

#### Size of the ILP

For each CFMRD , the local level of the ILP assigns a constant number of variables to each vertex and edge (see Table 6). Additionally there is a constant number of constraints associated with each vertex and edge (see Tables 1, 2, 3, 4). For each edge in the phylogeny, there is a constant number of constraints and variables per edge or vertex in the global level (see C.01 to C.03 and Table 5 respectively). The size of the ILP is thus linear with respect to the total size of all CFMRDs of the tree.

### Pre-processing

We provide two pre-processing options aimed at reducing the solution space. Firstly, we give the option to calculate an initial solution the solver starts with – guaranteeing that an approximate solution will be found, but also providing an immediate upper bound on the problem. The algorithm to compute such a solution proceeds in two steps, corresponding to the global and local level of the ILP respectively. In the first step, the algorithm determines linearizations for all ancestral genomes using the algorithm described in Section 3.2, taking into account the weights of the adjacencies. As a second step, decompositions for each CFMRD are determined by greedily fixing cycles in order of ascending length in the graph.

The second option for pre-processing allows us to bound the solution from below by using knowledge not available to the solver. To see how this method works, consider two genomes $\mathbb {A},\mathbb {B}$ and a degenerate genome $\mathbb {D}$. Transforming $\mathbb {A}$ into a linearization of $\mathbb {D}$ and this linearization into $\mathbb {B}$ must use at least as many DCJ- and indel-operations as transforming $\mathbb {A}$ into $\mathbb {B}$ via any intermediate genome $\mathbb {C}$ with the same copy-numbers of families as $\mathbb {D}$. This idea can be generalized to multiple intermediate genomes. Thus, by precomputing the distance $d(\mathbb {A},\mathbb {B})$ between leaves using ding [6] while taking into account the number of occurrences per family, we can derive the following additional global constraint:

$$\begin{aligned} (\texttt {C.opt}) \sum _{\begin{array}{c} E \in E(T)\\ E \text { on path between }\mathbb {A},\mathbb {B} \end{array}} {\texttt {f}}_E \ge d(\mathbb {A},\mathbb {B}) \quad \text { for all pairs of leaves }\mathbb {A},\mathbb {B}. \end{aligned}$$

## Evaluation

We implemented Algorithm 1 and made it publicly available^1^. We refer to this algorithm as *SPP-DCJ-v2* in the following. We performed a number of different experiments evaluating the solving time under different conditions as compared to SPP-DCJ as well as precision and recall for the safer linearization mode.

While solving the same problem, SPP-DCJ adds another parameter $\beta$ to the optimization which gives further negative weight to telomeres. In short, the optimization function of SPP-DCJ is equivalent to the form

Minimize

$$\begin{aligned} \alpha '\sum _{E\in E(T)} {\texttt {f}}_E \, + \, \beta ' \sum _{E\in E(T)} \# \text {telomeres in decompositions of }E \, - \, (1-\alpha ' - \beta ') \sum _{E\in E(T)} {\texttt {w}}_E \end{aligned}$$

We can simulate this behavior in our ILP by decreasing the assigned weight of telomeric adjacencies and by using a re-scaled $\alpha$.

When comparing to SPP-DCJ, we thus used default settings for SPP-DCJ with $\alpha '=\frac{1}{2}$, $\beta '=\frac{1}{4}$. This corresponds in our ILP to $\alpha =\frac{2}{3}$ and reducing the weight of each telomeric adjacency by 1, so we used these parameters for SPP-DCJ-v2 when comparing to SPP-DCJ.

We used gurobi version 12.0.0 on a single thread and with a time limit of 1 hour (3600 seconds) to solve the ILPs unless specified otherwise.

### Performance on linear genomes

In order to compare the behavior of SPP-DCJ and SPP-DCJ-v2 in the presence of multiple linear chromosomes, we used the simulator ffs-dcj introduced in [6]. The simulator performs a number of DCJs, indels and duplications with fixed rates for a given tree topology. In this experiment, we used a fixed balanced tree topology, namely (((*A* : 1.0, *B* : 1.0)*F* : 1.0), ((*C* : 1.0, *D* : 1.0)*G* : 1.0))*Root*; . We simulated 30 operations per branch on genomes of size 100 markers. More detailed settings (such as rates of duplications and indels) can be found in Table 7. The experiment was run for 2, 4, 6, 8, 10, 12, 14 and 16 linear chromosomes at the root of the tree with 10 replicates for each step. We then proceeded to introduce 30 adjacencies of adversarial noise for each sample at the inner nodes utilizing a script provided by the SPP-DCJ repository.

**Table 7:** Parameters for ffs-DCJ for the linear chromosome experiment

Duplication rate	0.4
Zipf parameter duplication	6.0
Deletion Rate	0.2
Insertion Rate	0.1
Zipf parameter indel	4.0

**Fig. 6:**
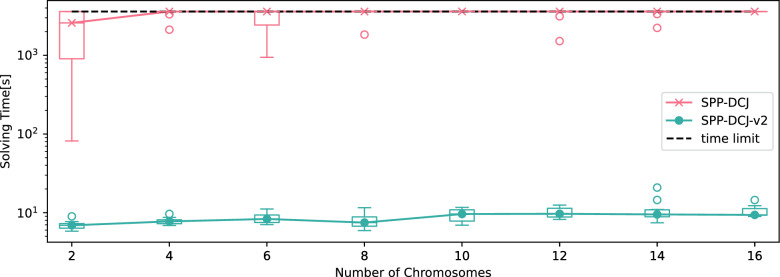
Solving times for SPP-DCJ and SPP-DCJ-v2 on simulated genomes with increasing numbers of telomeres. Solid lines represent corresponding median values

We then ran SPP-DCJ and SPP-DCJ-v2 on degenerate genomes consisting of the true and noise adjacencies. In order to ensure a fair comparison, we did not perform the performance optimizing pre-processing steps from Section "Pre-processing" or give any ranges for marker multiplicities in ancestral genomes for SPP-DCJ-v2. The results in solving time are shown in Fig. 6.

We see that SPP-DCJ-v2 on average needed more than two orders of magnitude less solving time than SPP-DCJ and even comparing the best run of SPP-DCJ to the worst of SPP-DCJ-v2 per step, the difference is still about one order of magnitude.

A majority of SPP-DCJ runs did not complete within the time limit. The performance of SPP-DCJ also dramatically worsens with increasing numbers of linear chromosomes, such that no ILPs were solved within the time limit for 16 chromosomes.

SPP-DCJ-v2 in turn was also affected by the rising numbers of linear chromosomes, but the effect is less drastic. In fact, the solving time for SPP-DCJ-v2 is well below a minute for all samples.

### Performance on circular genomes

As we have seen in Section "On linearizability", even when in the ground truth all linearizations of chromosomes are circular, additional telomeres might still be necessary to ensure that all degenerate genomes are linearizable.

**Fig. 7:**
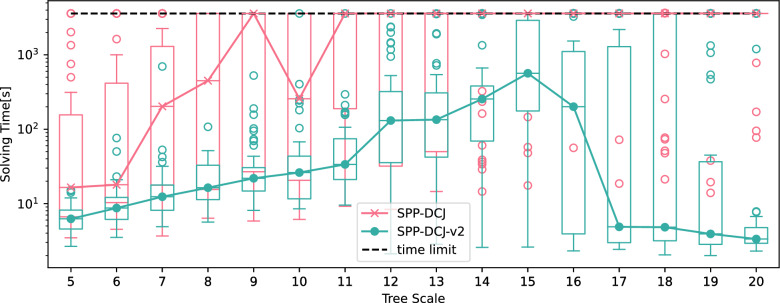
Solving times for SPP-DCJ and SPP-DCJ-v2 on genomes generated by ZOMBI on a range of trees with increasing branch lengths with ancestral adjacencies inferred by DecoSTAR. Solid lines represent corresponding median values

In order to examine this effect, we used the same pipeline as in [4] to simulate trees and genomes of 100 markers for each tree using ZOMBI [15] with tree scales ranging from 5 to 20 with 50 samples per step (for all parameter settings see Table 8). We then inferred degenerate genomes using DeCoSTAR [14] and solved the resulting SPP instances using SPP-DCJ and SPP-DCJ-v2, the latter again without additional pre-processing. We visualize the resulting solving times in Fig. 7.

**Table 8:** Parameter settings for ZOMBI and DeCoSTAR for the tree scale and precision experiments. For the sake of benchmarking SPP-DCJ-v2, ZOMBI parameters for genome evolution were chosen to represent an elevated degree of genome evolution, both in terms of gene content innovation (duplication+loss) and rearrangement (inversion+transposition)

	ZOMBI
DUPLICATION	f:2
INITIAL_GENOME_SIZE	100
LOSS	f:2
LOSS_EXTENSION	g:0.8
ORIGINATION	f:0
INVERSION	f:2
INVERSION_EXTENSION	g:0.5
TRANSPOSITION	f:2
TRANSPOSITION_EXTENSION	g:0.5
	DeCoSTAR
use.boltzmann	1
boltzmann.temperature	1.0
nb.sample	1000

Genomes generated by ZOMBI are circular, so one might assume that there is only negligible difference in runtime between SPP-DCJ and SPP-DCJ-v2. However, the results indicate that the improved handling of the solution space by SPP-DCJ-v2 allows it to solve problem instances with up to twice the tree scale as SPP-DCJ with comparable solving times.

Unexpectedly, the median solving times of SPP-DCJ-v2 decrease after a maximum at tree scale 15. We conjecture that this might be because enough rearrangements accumulate to make the genomes behave as if not related, making the problem easier to solve. SPP-DCJ however seems not to benefit from this effect in the tested tree scale and time range as the median solving time reaches the time limit for a tree scale of 11 and does not recover.

### Evaluation of the safer linearization mode

We used the same pipeline to simulate genomes of 1000 markers with ZOMBI, inferring degenerate ancestral genomes with DecoSTAR over a range of tree scales with five samples per step. All other parameters are the same as in Table 8. This time, however, we used SPP-DCJ-v2 with both the default and the safer linearization modes and examined the precision and recall of recovered adjacencies. In this experiment, we used $\alpha =0.5$ with weight 0 for the telomeric adjacencies added to ensure linearizability (see Section "On linearizability").

**Fig. 8:**
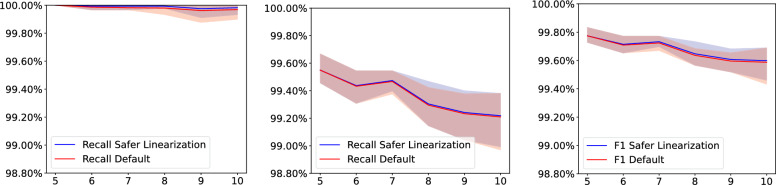
Mean precision, recall and F1 score for default and safer linearization mode for varying tree scales. Transparent ranges indicate minimum to maximum range of the five tested samples per step

The results, illustrated in Fig. 8, indicate that while our method displays very high precision and recall rates in both modes, the safer linearization mode has a minor, but consistent advantage over the default setting, especially considering precision. The trend in the data shows that this gap could widen further on more noisy data.

### Evaluating the Effect of Initial Solution and Lower Bounds

**Fig. 9:**
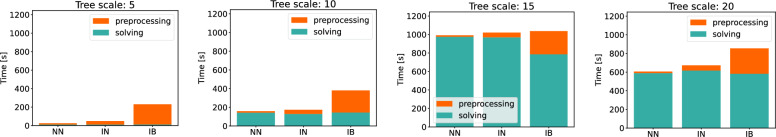
Average pre-processing and solving times of 50 samples for variants of SPP-DCJ-v2. NN - no additional pre-processing, IN - initial solution precomputed, IB - initial solution and lower bounds precomputed

We ran the ZOMBI pipeline again with the parameters detailed in Table 8 for tree scales 5, 10, 15,  and 20, generating 50 samples each. This time, we ran only SPP-DCJ-v2 and examined the effect of providing an initial solution or lower bounds for the ILP. The average solving and pre-processing times are given in Fig. 9.

While there is a slight trend in decreasing solving times with additional pre-processing, the time needed to apply the pre-processing itself dominates much of the runtime, especially on lower tree scales. Even on high tree scales, the benefit for the solving time seems to be outweighed by the time needed to complete the pre-processing. We therefore do not recommend computing an initial solution, unless no approximate solution would be found otherwise. In the same vein, we do not recommend precomputing lower bounds, unless they were already used to construct the phylogeny. Possibly the precomputation of lower bounds could be improved by only precomputing distances for certain pairs of leaves, and not all of them, which decreases the runtime while possibly keeping the positive effects on solving time.

### Reconstructing the ancestral X chromosomes of seven mosquitos

**Fig. 10:**
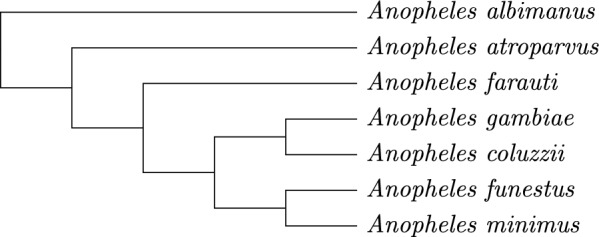
Cladogram for seven *Anopheles* taxa

We further evaluated our method on biological data from seven *Anopheles* species whose inferred phylogeny is depicted in Fig. 10. Gene annotations from protein coding genes of the X chromosome of present-day mosquitos were obtained from VectorBase [16]. Chromosome sizes fluctuated at around 600 genes. We then used the *ancestral gene order* (AGO) pipeline [8] to obtain candidate ancestral adjacencies. Using AGO, multiple sequence alignments were computed with MACSE [17], based upon which gene trees were inferred and reconciled with the species tree with IQ-TREE [18]. Finally, candidate ancestral adjacencies were computed with DeCoSTAR.

We ran SPP-DCJ and SPP-DCJ-v2 with varying optimization levels to generate corresponding ILPs and initial solutions where applicable. Additionally, we generated an ILP based on SPP-DCJ-v2, for which we allowed copy numbers in ancestral families to deviate by one from the maximum number of copies. The resulting ILPs were then input to gurobi 12.0.0, which ran on 10 threads with a time limit of 12 hours on the same machine for all ILPs.

**Fig. 11:**
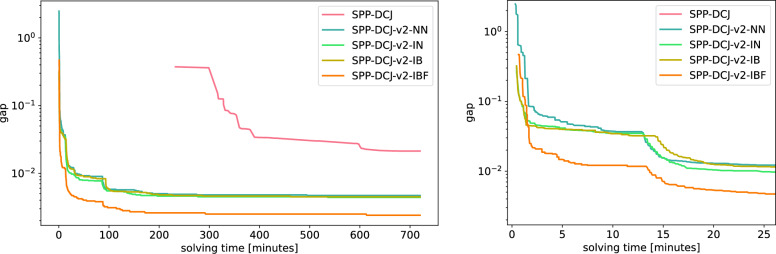
Gaps reported by gurobi with increasing solving times for SPP-DCJ and variants of SPP-DCJ-v2 until a time limit of 720 minutes. Right: Zoomed in on the first 25 minutes. NN - no additional pre-processing, IN - initial solution precomputed, IB - initial solution and lower bounds precomputed, IBF - initial solution and lower bounds precomputed, with variable ancestral family sizes

We visualize the gaps reported by gurobi over time in Fig. 11. For all variants of SPP-DCJ-v2 gurobi found solutions with significantly closer gaps than for SPP-DCJ. In fact, results as close as the final result for SPP-DCJ were found for all versions of SPP-DCJ-v2 within the first 25 minutes of solving time.

As before, the pre-processing optimizations have only a minor effect on the quality of the result at the end of solving time. Indeed, the effect is most strongly visible within the first few minutes of solving time, after which gurobi’s own heuristic solutions start to overshadow the initial solutions found in pre-processing.

Interestingly, allowing for uncertainty about the multiplicity of the families in ancestral genomes did not slow computation, but had an immense speed up effect. This suggests that doing so allows to find solutions better fitting the given phylogeny and adjacencies.

## Discussion

We presented SPP-DCJ-v2, the first ILP of polynomial size to solve the Small Parsimony Problem for natural genomes under the DCJ-indel model. Using a more efficient representation of the solution space, the Capping-Free Multi-Relational Diagram, we were able to significantly improve upon the performance of its predecessor, SPP-DCJ. Additionally, we introduced a new method of ensuring linearizability that is more robust when applied to (potentially noisy) real data because linearization is not the main constraint any more. We also introduced a feature that allows users to specify their own bounds on marker multiplicities in ancestral genomes, which may help in ambiguous cases on real data. We evaluated our method on simulated data and found it to be more efficient than its predecessor. Additionally bounds on the solution space do not seem to help performance, especially when considering the additional time needed for pre-processing, but could potentially be helpful on very large problem instances. Finally, we demonstrated that our approach is efficient enough to derive good solutions for SPP on real phylogenies within reasonable time frames.

## Data Availability

No datasets were generated or analysed during the current study.

## Footnotes

1 https://github.com/codialab/spp-dcj and can be installed with conda install -c conda-forge -c bioconda spp-dcj
